# Merciful Malady: Renbök Phenomenon Between Alopecia Areata and Psoriasis Vulgaris

**DOI:** 10.7759/cureus.99826

**Published:** 2025-12-22

**Authors:** Ramya A, Adikrishnan Swaminathan, Arun Vignesh, Ambigai SSK, Sudha Rangarajan

**Affiliations:** 1 Dermatology, Venereology and Leprosy, Sri Ramachandra Institute of Higher Education and Research, Chennai, IND

**Keywords:** alopecia areata, inverse koebnerisation, psoriasis vulgaris, renbok phenomenon, sparing phenomenon in dermatology

## Abstract

RenbÖk phenomenon is the withdrawal of one clinical lesion when another lesion appears. This is a case report of an 11-year-old girl with multiple hyperpigmented scaly plaques over the scalp, trunk, and bilateral upper and lower limbs. The patient also had patchy hair loss over the scalp, which was spared from the scaly plaques. The case report thus elaborates on a fascinating immunological mechanism between the two autoimmune diseases that spares the patient from one disorder should the other appear.

## Introduction

Described by Happle et al. in 1991, the RenbÖk phenomenon (RP) is used to describe the withdrawal of one lesion at the onset of another [1]. Initially, it was described in alopecia areata (AA) patients experiencing hair growth when psoriatic plaques appeared on the areas of hair loss [1]. While lesions are provoked in response to trauma in Koebner's phenomenon, RP shows the inhibition of a particular inflammatory response and is hence called inverse Koebnerization [2]. This sparing effect reveals the unique immunological antagonism between the two conditions [3]. Thus, understanding this phenomenon will contribute to broader knowledge on immune privilege, disease co-existence, and potential therapeutic synergies. In this case report, we describe a patient in whom an AA patch was spared from the psoriatic lesions.

## Case presentation

A 11-year-old girl was brought to the dermatology OPD with complaints of multiple scaly lesions all over the body for the past 3 months.

The patient was apparently normal prior to three months, after which she started developing a few dark, raised lesions over the bilateral knees, followed by the elbows. The lesions slowly progressed to involve the bilateral upper limbs, lower limbs, and trunk. She had a history of developing scaling over the scalp that gradually progressed to form large confluent lesions that covered most of the scalp's surface. A history of developing patchy loss of hair over the scalp was also present.

The patient had no history of joint pain or any suggestive of any focus of infection. There was no history of topical or oral native medication use or topical irritant use, nor a history of atopy or history of pulling of hair. A dermatological examination of the patient revealed the findings listed in Table 1.

**Table 1:** Various findings noted in this patient

Disease	Clinical findings	Bedside tests
Psoriasis vulgaris	Hyperpigmented scaly papules and plaques over the scalp, trunk, and bilateral upper and lower limbs	Grattage test showed a positive Auspitz sign.
Alopecia areata	Smooth patch of hair loss noted over the frontal region of the scalp, devoid of scaly plaques	The hairpull test was negative. Dermoscopy showed white dots, broken hair, and vellus hair with scaly plaques ending sharply at the border of the patch.

**Figure 1:**
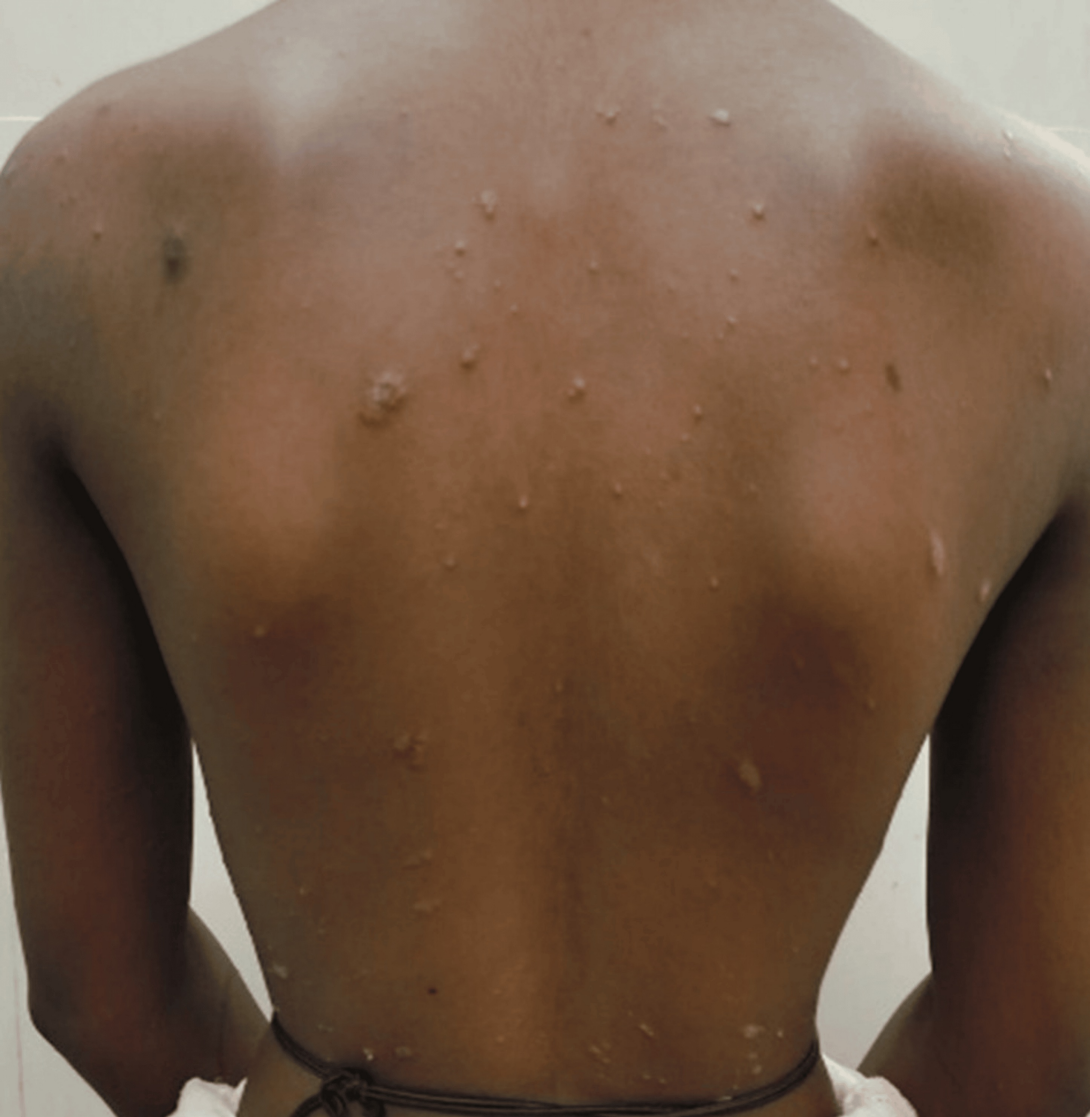
Multiple scaly papules and plaques over the trunk

**Figure 2:**
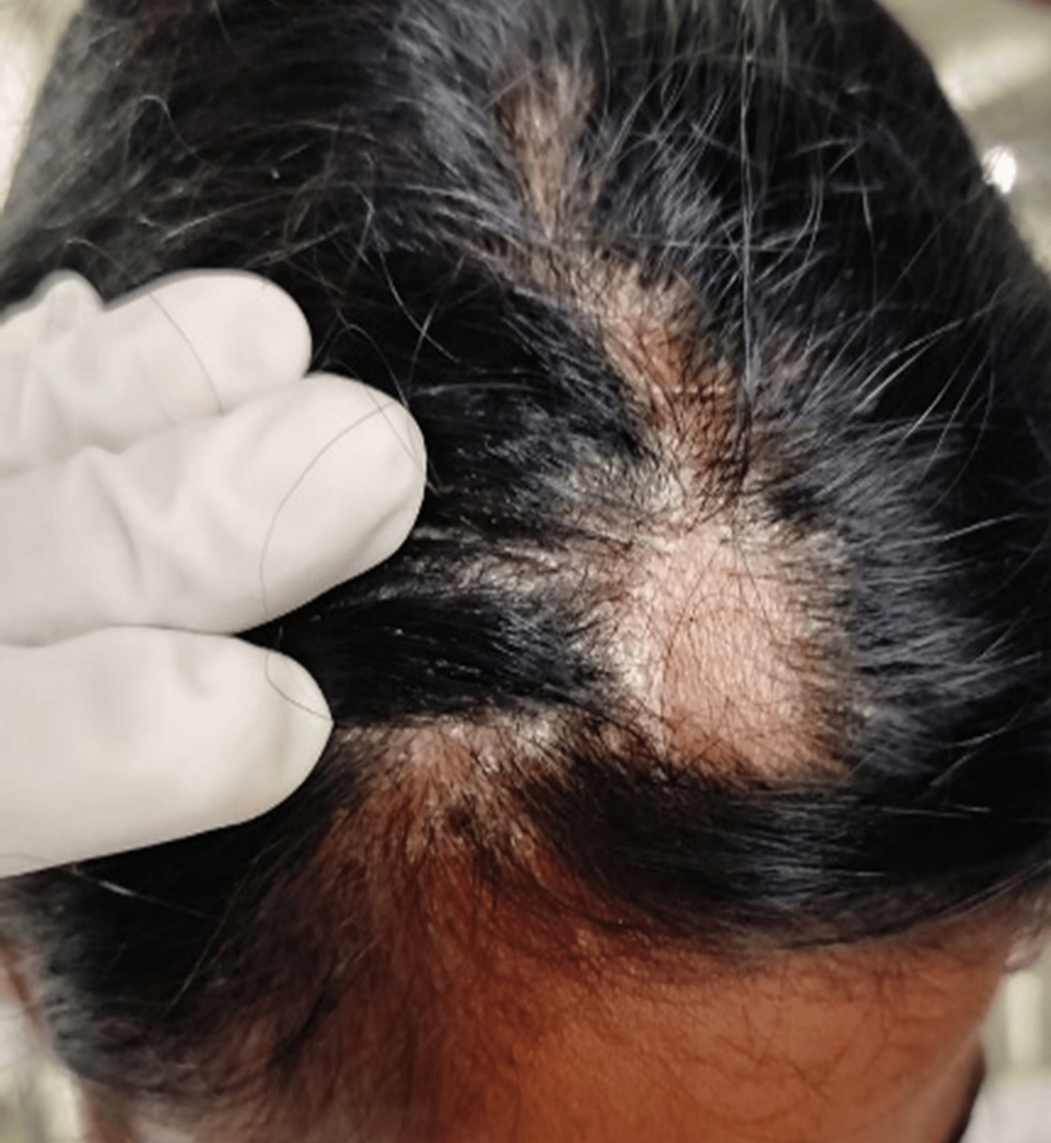
Psoriatic plaques, which initially started as papules, and later evolved to form multiple large plaques on the scalp, sparing the alopecia areata patch

**Figure 3:**
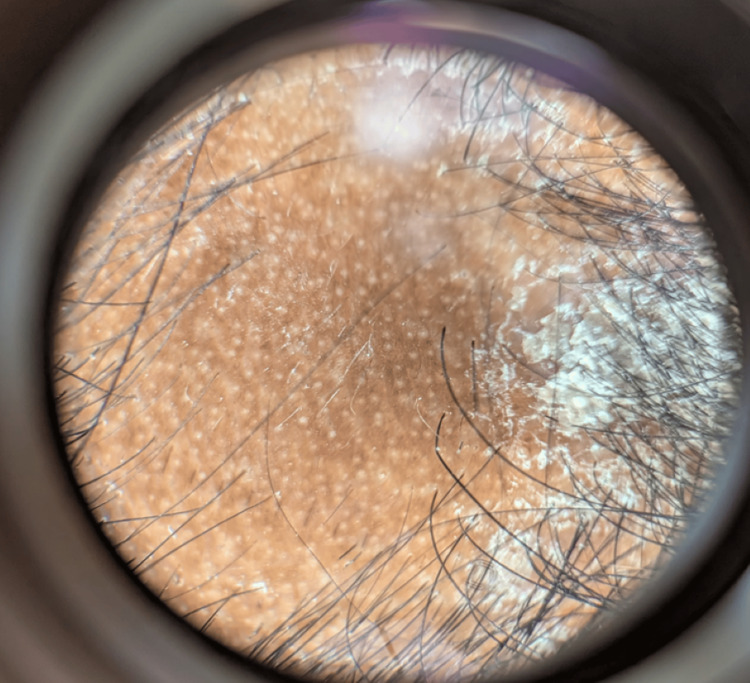
Dermoscopy showing white dots, vellus hair, and the sharp demarcation between the alopecia areata patch and psoriatic plaques

The patient had multiple hyperpigmented scaly papules and plaques over the trunk, bilateral upper and lower limbs, and scalp (Figure 1). A single smooth patch of hair loss was noted over the frontal area of the scalp, devoid of scaling, and surrounded by a sharp demarcation where the psoriatic plaques ended at the margins (Figure 2). Nails showed coarse pitting while the palms and soles were normal. The Grattage test was done over the truncal scaly plaques, which showed a positive Auspitz sign. The hair pull test was negative. Dermoscopic findings of the AA patch showed white dots, broken hair, and vellus hair with scaly plaques ending sharply at the border of the patch (Figure 3).

Routine investigations were done to establish a baseline and to rule out infective foci. Considering the efficacy and safety profile in this age group, the patient was started on Tab. Tofacitinib 5 mg HS, along with a topical steroid, with minoxidil for alopecia areata and topical emollients with steroids for the psoriatic lesions.

## Discussion

The term RenbÖk was coined as a reverse spelling of Koebner, highlighting the conceptual opposition. Though originally observed between AA and psoriasis, over the years, it has also been noted between AA and nevus flammeus and vitiligo. There have been other case reports of RP where drug eruptions spared seborrheic keratosis, psoriatic plaques, nevus depigmentosus, and guttate psoriasis, which spared Becker melanosis [4-7].

Autoimmune disorders of childhood deserve a special mention, considering the long-term implications of both the disease per se and the effects of the treatment modalities. While psoriasis vulgaris is one of the most common papulosquamous disorders in childhood, AA is also frequently encountered. Recent advances in the pathogenesis have identified that different T cell populations are at play in each of these disorders [8]. This case report is about a patient with colocalization of both disorders, making it peculiar. The interaction between these dermatological diseases offers insight into the immunological dynamics of both.

AA is a non-scarring alopecia caused by an immune response mounted against a hair follicle. The primary pathogenesis begins after the loss of the immune privilege of anagen hair follicles, following which there is a Th-1-mediated inflammatory response against the hair follicle. Cytokines like IFN-γ, TNF-α, IL-2, IL-12, and IL-17, and several chemokines, such as CXCL9 and CXCL10, play a major role in its pathogenesis [9]. These chemokines attract cytotoxic T cells, minimizing Th-17 recruitment and thus explaining the clearance of psoriatic lesions on an alopecia patch. Psoriasis vulgaris is a Th-17 disease that results in keratinocyte proliferation, leading to epidermal thickening and plaque formation. Cytokines like IL-17A, IL-23, IFN-γ, and TNF-α play key roles in the pathogenesis of this disorder [10].

The exact pathogenesis of RP is still under study. The primary understanding is that when both of these conditions coexist, the innate nature for one inflammatory response to dominate by inhibiting the other makes one disease regress. The difference in underlying immune mechanisms leads to changes in the local cytokine milieu, resulting in this rare phenomenon [11,12]. The factor that decides on the presiding inflammatory response remains an enigma.

Apart from the obvious differences in cytokines, neuropeptides have also been found to play a part in this phenomenon. Studies have shown that there are reduced levels of substance P in late AA patches along with reduced calcitonin gene-related peptide levels [13]. Both of these neuropeptides have been found to be released by nerve fibers, aiding in the formation of new psoriatic lesions by increasing keratinocyte proliferation. Their levels have been found to be higher in the serum of psoriatic patients [14]. Since these levels are reduced in the alopecia patches, they become a cold zone for psoriatic inflammation, justifying the absence of lesions.

While it's usual for AA to resolve, leading to terminal hair growth in hairless patches with psoriatic lesions, in this patient, we observed that while the alopecia persisted, psoriatic lesions spared the patch, making this case unique.

## Conclusions

RP thus represents a fascinating example of localized immune antagonism, which was most strikingly observed in this patient. Though both conditions are autoimmune in nature, the similarities end there. The varied Th-response and the difference in the cytokine milieu lead to a competitive environment where ultimately one pattern persists, leading to regression of the other. By shedding light on how inflammatory pathways interact, oppose, and dominate each other, comprehending this unusual phenomenon contributes to a deeper understanding of cutaneous immunology, helping us better understand the pathogenesis and treatment options of both these diseases.
